# Transformation of Pathology Reports Into the Common Data Model With Oncology Module: Use Case for Colon Cancer

**DOI:** 10.2196/18526

**Published:** 2020-12-09

**Authors:** Borim Ryu, Eunsil Yoon, Seok Kim, Sejoon Lee, Hyunyoung Baek, Soyoung Yi, Hee Young Na, Ji-Won Kim, Rong-Min Baek, Hee Hwang, Sooyoung Yoo

**Affiliations:** 1 Office of eHealth Research and Business Seoul National University Bundang Hospital Seongnam Republic of Korea; 2 Department of Pathology and Translational Medicine Seoul National University Bundang Hospital Seongnam Republic of Korea; 3 Division of Hematology and Medical Oncology Seoul National University Bundang Hospital Seongnam Republic of Korea; 4 Department of Plastic Surgery Seoul National University Bundang Hospital Seongnam Republic of Korea

**Keywords:** common data model, natural language processing, oncology module, colon cancer, electronic health record, oncology, pathology, clinical data

## Abstract

**Background:**

Common data models (CDMs) help standardize electronic health record data and facilitate outcome analysis for observational and longitudinal research. An analysis of pathology reports is required to establish fundamental information infrastructure for data-driven colon cancer research. The Observational Medical Outcomes Partnership (OMOP) CDM is used in distributed research networks for clinical data; however, it requires conversion of free text–based pathology reports into the CDM’s format. There are few use cases of representing cancer data in CDM.

**Objective:**

In this study, we aimed to construct a CDM database of colon cancer–related pathology with natural language processing (NLP) for a research platform that can utilize both clinical and omics data. The essential text entities from the pathology reports are extracted, standardized, and converted to the OMOP CDM format in order to utilize the pathology data in cancer research.

**Methods:**

We extracted clinical text entities, mapped them to the standard concepts in the Observational Health Data Sciences and Informatics vocabularies, and built databases and defined relations for the CDM tables. Major clinical entities were extracted through NLP on pathology reports of surgical specimens, immunohistochemical studies, and molecular studies of colon cancer patients at a tertiary general hospital in South Korea. Items were extracted from each report using regular expressions in Python. Unstructured data, such as text that does not have a pattern, were handled with expert advice by adding regular expression rules. Our own dictionary was used for normalization and standardization to deal with biomarker and gene names and other ungrammatical expressions. The extracted clinical and genetic information was mapped to the Logical Observation Identifiers Names and Codes databases and the Systematized Nomenclature of Medicine (SNOMED) standard terminologies recommended by the OMOP CDM. The database-table relationships were newly defined through SNOMED standard terminology concepts. The standardized data were inserted into the CDM tables. For evaluation, 100 reports were randomly selected and independently annotated by a medical informatics expert and a nurse.

**Results:**

We examined and standardized 1848 immunohistochemical study reports, 3890 molecular study reports, and 12,352 pathology reports of surgical specimens (from 2017 to 2018). The constructed and updated database contained the following extracted colorectal entities: (1) NOTE_NLP, (2) MEASUREMENT, (3) CONDITION_OCCURRENCE, (4) SPECIMEN, and (5) FACT_RELATIONSHIP of specimen with condition and measurement.

**Conclusions:**

This study aimed to prepare CDM data for a research platform to take advantage of all omics clinical and patient data at Seoul National University Bundang Hospital for colon cancer pathology. A more sophisticated preparation of the pathology data is needed for further research on cancer genomics, and various types of text narratives are the next target for additional research on the use of data in the CDM.

## Introduction

Colorectal cancer is the third most common cancer in the world after lung cancer and breast cancer, and the second most common cause of cancer deaths in the world after lung cancer [1]. In addition, the incidence of colorectal cancer in Korea is continuously increasing owing to the westernization of diet and the widespread use of colonoscopy [2]. To determine treatment and prognosis, clinical and pathologic staging are both crucial. Pathology reports vary in format worldwide. The heterogeneity of pathology reports is not unique to colorectal cancer; there has been a growing need to standardize pathology reports [3]. Pathologic diagnosis is based on gross examination, microscopic examination, and sometimes molecular testing. Although some hospitals report molecular testing results in a structured format, biomarker results in the pathology report are usually recorded as unstructured free text or template-based semistructured text in electronic health record (EHR) systems. This unstructured document must be converted into a structure that can be processed by a computer.

Unstructured clinical narratives may summarize patients’ medical history, diagnoses, medications, immunizations, allergies, radiology images, and laboratory test results in the form of progress notes, discharge reports, etc, providing a valuable resource for computational phenotyping [4]. Previous studies have applied controlled vocabularies such as the Systematized Nomenclature of Medicine Clinical Terms (SNOMED CT) collection to recognize various expressions of the same medical concepts in pathology and used the Unified Medical Language System (UMLS) as a metathesaurus [5-7]. Other significant efforts have been devoted to the implementation of open-source, standards-based systems to improve the portability of EHR-based phenotype definitions (eg, eMERGE [Electronic Medical Records and Genomics] [8] and PhEMA [Phenotype Execution and Modeling Architecture] [9]).

Common data models (CDMs) are healthcare data models with a standard structure. An example is the Observational Medical Outcomes Partnership (OMOP) CDM, adopted and maintained by Observational Health Data Sciences and Informatics (OHDSI) [10-13]. OHDSI is an open-science community that aims to improve health by empowering the community to collaboratively generate evidence that promotes better health decisions and care [14]. OHDSI conducts methodological research to establish scientific best practices for the appropriate use of OMOP CDM data and develops open-source analytics software for research use. The OHDSI oncology working group incorporated fundamental structural and semantic support into the OMOP CDM to represent clinical cancer disease and treatment data, significantly improving the specificity of cancer cohort definitions. To represent cancer diagnoses using the combination of histology and topography in the OMOP CDM Condition domain (Condition_Occurrence), without changes to the existing structure, they have proposed a precoordinated collapse of the International Classification of Diseases for Oncology (ICD-O) axes—histology and topography—to a single OMOP-originated concept representing a unique cancer diagnosis, which preserves linkages between these single codes and the ICD-O axes in the OMOP standard vocabulary.

To transform EHR data into the OMOP CDM format, data formalization and vocabulary mapping should be performed in advance. Thus, most structured clinical EHR data, such as diagnosis, medications, lab tests, and vital signs, are usually converted to the CDM first. However, a large amount of data in EHRs—including pathology reports—is recorded and stored in an unstructured or semistructured form. In particular, it is essential to extract and refine major clinical entities from the pathology reports through natural language processing (NLP) to utilize both clinical and genomics data in CDM-based cancer research.

In this study, we transformed and incorporated the colorectal cancer pathology reports of a tertiary general university hospital into the OMOP CDM format by developing an NLP module. The primary objective of this research is to (1) extract major biomarker entities from the pathology report, (2) convert them into the OMOP CDM format by mapping the vocabulary to standardized terminology, and (3) demonstrate the ability of the OMOP CDM to represent biomarker data using the OMOP CDM oncology extension module.

## Methods

### Study Data

The study site, Seoul National University Bundang Hospital (SNUBH), has converted EHR data from a 15-year period—from the opening of SNUBH until December 2018—into the OMOP CDM format. The CDM database contains data on more than 1.7 million patients. Although patient data such as basic demographic information, medical history, family history, diagnosis, drug exposures, test results, vital signs, surgeries, and procedures were converted into the OMOP CDM standard, free-text results, such as pathology reports, are stored in the form of free text in a table called NOTE. It is necessary to extract these data from the reports through text processing techniques for use in clinical research. In this study, the pathology reports of patients diagnosed with colorectal cancer from 2017 to 2018 were processed to investigate the ability of the OMOP CDM Oncology module to represent three types of pathology reports: pathology reports of surgical specimens, immunohistochemical study reports, and molecular study reports. This study was approved for exemption by the SNUBH Institutional Review Board.

**Figure 1:**
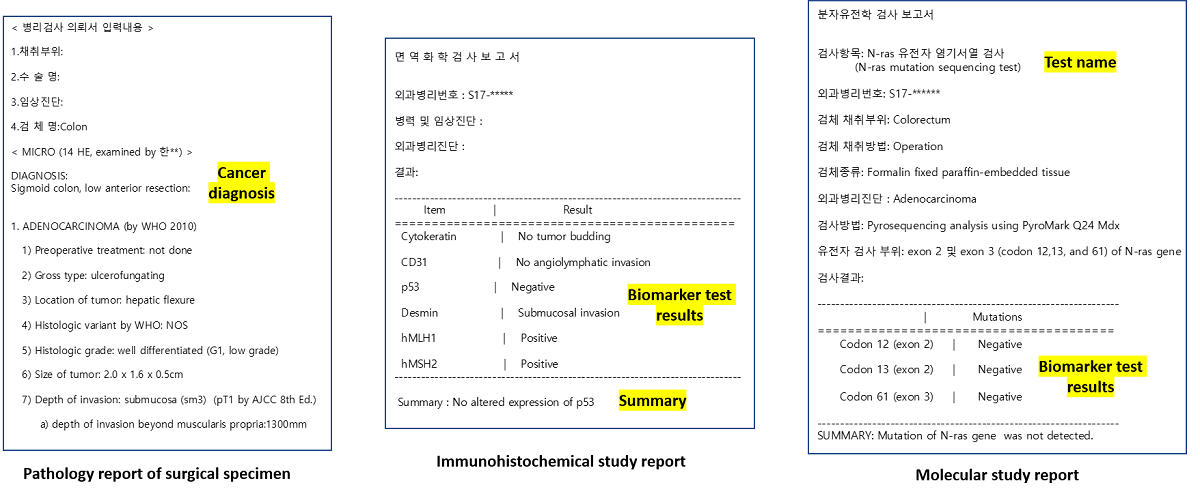
Contents of information to extract by report type.

The original data were derived from the NOTE table of the SNUBH OMOP CDM database and included the original free-text data of pathology reports of surgical specimens, immunohistochemical study reports, and molecular study reports (Figure 1). Surgical number, biomarker name, test result, and summary information were extracted from the immunohistochemical study tests. In pathology reports, a molecular study is a method of analyzing protein expression in cells; in this study, surgical number, test name, gene name, mutation examination result, and summary information were extracted from molecular study tests. The surgical pathology report is an essential part of patient care because it documents the pathologic findings in tissues removed from patients for diagnostic or therapeutic reasons [15]. Diagnosis names were extracted from pathology reports of surgical specimens.

### Processing Model for Rule-Based Text Extraction

**Figure 2:**
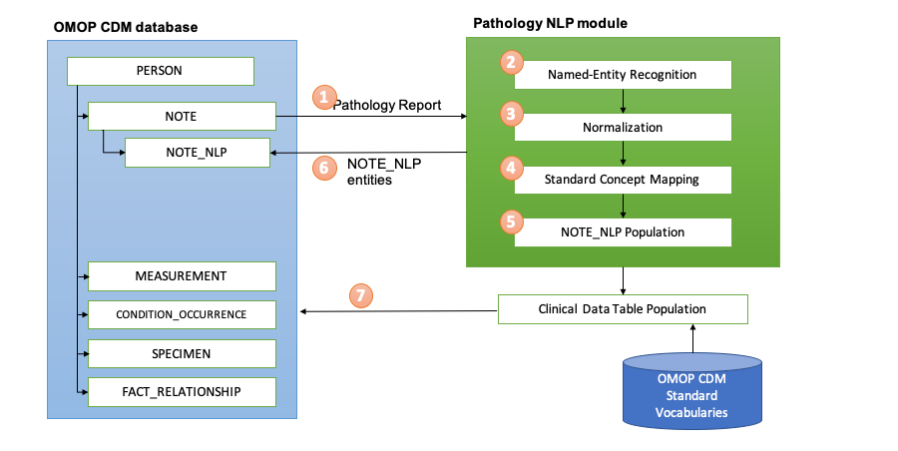
Overall process of this study. CDM: common data model. NLP: natural language processing. OMOP: Observational Medical Outcomes Partnership.

The types of key information recorded in pathology reports vary based on the pathological tests conducted. We first reviewed the format and text entities of the extracted original report and selected the target entities and values for information extraction. Rule-based text processing based on regular expressions was developed to extract the clinical entities of the test subject and result information depending on the type of report. In the immunohistochemical study reports and molecular study reports, target gene or protein information and the test result values were recognized and extracted, whereas pathology reports of surgical specimens were processed for information on the diagnosis and the region that was operated on. This rule-based approach is ideally suited for the retrieval of unstructured pathologic clinical entities and test result values in the SNUBH EHR notes. Figure 2 shows the NLP module developed in this study and depicts the overall study process, with the numbers in the circles indicating the sequence of steps.

The measurement value extraction system was packaged through a regular expression–based Python NLP pipeline using a method similar to that employed in previous research [16]. Our system recognizes clinical entities such as the biomarkers in a test and their values. Our system performs three broad steps: (1) it identifies the measurement name, such as gene or protein name, in the text; (2) it identifies the measurement value in the text; and (3) it transforms the appropriate value expressions according to their relationship. For immunohistochemical and molecular study tests, rules were created for extracting the target gene name, protein name information, and test result value. For example, surgical number, biomarker name, test result, and summary information were extracted from the immunohistochemical study tests (example in Figure 1). Text entities for a test result could include expressions such as “EGFR (GI)” (epidermal growth factor receptor; gastrointestinal) and “1+/3.” The term “EGFR (GI)” is regularized into “EGFR” and “epidermal growth factor receptor Ag [presence] in tissue by immune stain”. Through this clinical entity recognition, we could structure data into tables that can be transformed into the NOTE_NLP table after term standardization in the CDM (Table 1 and Figure 1).

Several modifiers were added to enter data extracted from the NOTE_NLP table. For the immunohistochemical study report and the molecular study test report, the raw text of the test results was entered in the value_as_narrative column, and the information on whether the normalized value was a negation expression and the concept_id were included in the value_as_concept_id column. The names of each report were added to the section_source_value column, and inspection item information was included for molecular study test reports. In addition, the surgical pathology number was added to the sub_id column to be linked to surgical specimen pathology reports.

We used Python version 3.6 and regular expressions to process three types of colon cancer pathology reports according to each report’s characteristics. For example, the specimen and diagnosis names were extracted from surgical specimen pathology reports, with the specimen name written in each report as “SAMPLE NAME: colon,” using a regular expression to extract the items after “SAMPLE NAME:.” Likewise, the diagnosis name is found after the entity expression “DIAGNOSIS:.” From the immunohistochemical study report, items following “SUMMARY:” and prior to “EXAMINER:” were extracted as summary information. In addition, biomarker results from the immunohistochemical study and molecular study pathology reports were extracted in a semistructured text table with continuous values such as hyphens (-). To deal with this text, data fields were separated based on the continuous value of hyphen (-), with biomarker name on the left of the vertical bar (|) and result value on the right. Refer to Multimedia Appendix 1 for processing rules (Table S1).

**Table 1:** NOTE_NLP table for pathology report data.

NOTE_NLP field	Surgical pathology report	Immunochemistry report	Molecular study report
NOTE_NLP_ID	12345	23456	34567
NOTE_ID	1	2	3
SNIPPET	ductal adenocarcinoma, moderately differentiated > poorly differentiated	EGFR (GI) | 1+/3	Codon 12 (exon 2) | Positive
OFFSET	560,632	170,188	200,231
SECTION_CONCEPT_ID	3025891 (Pathology report final diagnosis narrative)	40758358 (Immune stain study)	3001274 (NRAS gene mutations found [identifier] in blood or tissue by molecular genetics method nominal)
LEXICAL_VARIANT	Adenocarcinoma	EGFR	Codon 12 (exon 2)
NOTE_NLP_CONCEPT_ID	0	3016231 (Epidermal growth factor receptor Ag [presence] in tissue by immune stain)	0
NOTE_NLP_SOURCE_CONCEPT_ID	44498791 (Tubular adenoma, NOS^a^)	0	0
TERM_MODIFIERS	Negated=FALSE; sub_id=S 110023456; section_source_value=Pathology reports of surgical specimen	Negated=FALSE; value_as_concept_id=9191; value_as_narrative=1+/3; sub_id=S 120034567; section_source_value=Immunochemistry test report	Negated=FALSE; value_as_concept_id=9191; value_as_narrative=Missense mutation [c.38G>A, p.Gly14Asp]; sub_id=S 130045678; section_source_value=N-ras Gene sequencing test

^a^NOS: not otherwise specified.

### Vocabulary Standardization for Pathology Reports

To standardize the extracted pathologic data, protein or gene laboratory tests and pertinent test result values were mapped into the OMOP standard concept vocabulary and reviewed by a clinician, a nurse, and a bioinformatics expert. The test names were mapped to standard concepts of Logical Observation Identifiers Names and Codes (LOINC) in the MEASUREMENT domain, and the result values were normalized to those concepts (eg, categorized as positive or negative).

According to the OMOP CDM Oncology module, information on the cancer diagnosis and the region operated on, taken from the surgical specimen pathology reports, was mapped to the ICD-O, Third Edition, and then stored in the CONDITION_OCCURRENCE table with ICD-O-3 and its mapped SNOMED CT codes.

To map the extracted clinical and gene information to a standard concept_id, we constructed a dictionary table for the test names and result values that appear in the free-text pathology reports. For example, in immunohistochemical study reports, the test name for EGFR is concept_id 3016231 (Epidermal growth factor receptor Ag [presence] in tissue by immune stain), which is the LOINC code 32581-1 with a positive or negative result value. The result value of “1+/3” was normalized and mapped to the concept_id of 10828004 (Positive), which is SNOMED CT code 9191. The molecular study test report comprised results of four types of examinations: *NRAS* (OMIM 164790) mutation, *KRAS* (OMIM 190070) mutation, *BRAF* (OMIM 164757) mutation, and microsatellite instability. For each test result value, the normalization rules for test result values were separately defined and coded, as shown in Table 2.

**Table 2:** Defined mapping rules for OMOP CDM concept_id, LOINC code, and test results from the molecular study pathology reports.

Test name	CONCEPT_ID	LOINC code	RESULT_Attribute	Normalization rules for results value concept
NRAS mutation	3001274	21719-0	Codon 12 (exon 2), Codon 13 (exon 2), Codon 61 (exon 3)	If 1 or more positive values are present, treat as total positive
KRAS mutation	36203353	85509-8	Codon 12 (exon 2), Codon 13 (exon 2), Codon 61 (exon 3)	If 1 or more positive values are present, treat as total positive
MSI^a^	3047348	43368-0	BAT26, BAT25, D5S346, D17S250, D2S123	Categorized as MSI-H (high) if more than 1 value is positive, MSI-L (low) if 1 value is positive, and MSS^b^ if no positive value is present
BRAF mutation	40761583	58483-9	V600E, K601E	If more than 1 positive value is present, treat as total positive

^a^MSI: microsatellite instability.

^b^MSS: microsatellite stable.

A total of 78 biomarkers were used in immunohistochemical reports, most of which are measurement domains, and the concept_id for each was retrieved and mapped from the OMOP CDM. However, there were 3 undetected biomarkers that were used with their own concept_id (3/78, 4%). Refer to Multimedia Appendix 2 for a list of mapped concepts (Table S2).

After term mapping, the NOTE_NLP table of the CDM was derived. To integrate the extracted data into the existing CDM data, the main results of immunohistochemical and molecular study tests were updated in the MEASUREMENT table. In addition, for the cancer diagnosis from surgical specimen pathology reports, relevant information was added to the CONDITION_OCCURRENCE table. To define the relationship between the MEASUREMENT, CONDITION_OCCURRENCE, and SPECIMEN tables, we populated the CDM FACT_RELATIONSHIP table. With the two existing concepts, “Specimen source identity (SNOMED)” and “Has specimen (SNOMED),” we could define the target specimen and its attribute.

### System Validation

Two experts manually reviewed the transformed data in the NOTE_NLP table in 100 randomly selected pathology reports. To verify the accuracy of the extracted values for each table item, the nurse and medical information specialist manually checked the original pathology document and extracted text values. Furthermore, two reviewers manually reviewed the original document and checked the results of the processing rules.

## Results

### Major Text-Entity Extraction and Mapping Onto International Standard Terminology

**Figure 3:**
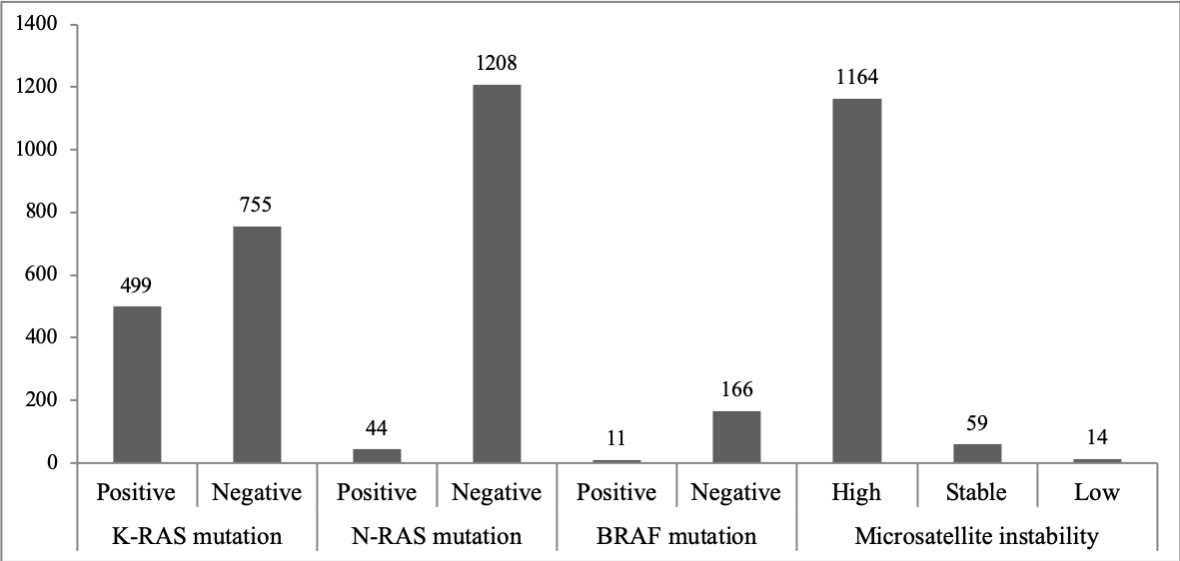
Each biomarker and its results extracted from molecular study pathology reports.

**Figure 4:**
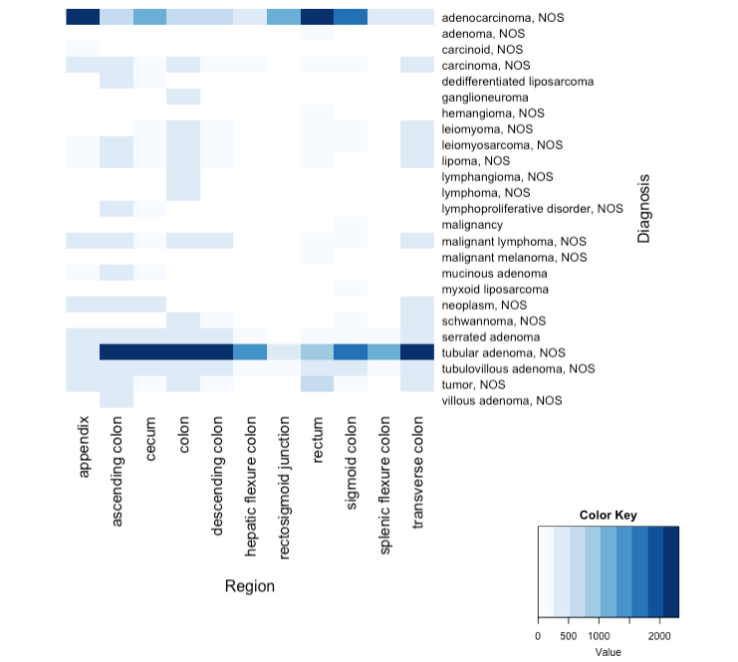
Frequency distribution in surgical pathology reports. NOS: not otherwise specified.

Pathologic clinical information on colorectal cancer was extracted through NLP of immunohistochemical studies, molecular studies, and surgical specimen pathology reports to construct a CDM-formatted NOTE_NLP table. NLP extraction and terminology standardization were performed for 1848 immunohistochemical study reports, 3890 molecular study reports, and 12,352 surgical specimen pathology reports. The number of items, such as protein or gene name values, extracted from the immunohistochemical study reports was 6092, and the number extracted from the molecular study reports was 13,953; the summarized essential information was then updated in the existing MEASUREMENT table. Cancer diagnosis entities from the surgical specimen pathology reports (25,902) were delivered to the CONDITION_OCCURRENCE table. Figure 3 shows the number of each biomarker extracted from molecular study pathology reports by result, and Figure 4 shows the frequency of each diagnosis name and its surgical regions as a heatmap plot. *KRAS* mutations, *NRAS* mutations, *BRAF* mutations, and microsatellite instability are tested in the hospital. For each test result value, the normalization rules for test result values were separately defined and coded as described in Table 2. The majority of the test results are “negative” or “microsatellite instability high.” In Table 3, we included evaluation results for each attribute extracted and transformed into standard terminology concepts for each pathology report. The results of the attribute value extraction task are quite good. The attributes derived from surgical specimen pathology and molecular study reports were recognized 100% correctly among randomly selected test reports. “No endolymphatic tumor emboli” or “no lymphoepithelial lesion” values were recognized as positive in a few immunohistochemical study entities. This is because we have created rules that focus on processing the term “negative” when we process biomarker text.

According to Figure 4, there were 11 colon surgical regions at SNUBH from 2017 to 2018, and the most frequent diagnosis names were “tubular adenoma, nos” (not otherwise specified) and “adenocarcinoma, nos.” Positive and negative biomarker results extracted from immunohistochemical study reports are shown in Table 4.

**Table 3:** Evaluation results for attributes found in 100 pathology test reports.

Type of report	Attribute example	Entities, N	Precision, n (%)	Recall, n (%)	F-measure
Surgical specimen pathology report	ductal adenocarcinoma, moderately differentiated > poorly differentiated	109	109 (100)	109 (100)	100
Immunohistochemical study	EGFR (GI) | 1+/3	367	362 (98.6)	367 (100)	99.31
Molecular study	Codon 12 (exon 2) | Positive	100	100 (100)	100 (100)	100

**Table 4:** Top 20 biomarker results from immunohistochemistry tests.

CONCEPT_ID	CONCEPT_NAME	Positive (N=4626), n (%)	Negative (N=2096), n (%)
3016231	EGFR	1360 (29.4)	33 (1.6)
21493968	hMLH1	361 (7.8)	25 (1.2)
3046605	Ki-67	359 (7.8)	0 (0.0)
21493983	PTEN	261 (5.6)	76 (3.6)
3017031	p53	111 (2.4)	212 (10.1)
21493982	BRAF	12 (0.3)	310 (14.8)
3027870	CD3	153 (3.3)	160 (7.6)
3019066	C-erbB2	75 (1.6)	194 (9.3)
3026213	CD20	171 (3.7)	37 (1.8)
21493969	hMSH2	185 (4.0)	18 (0.9)
3051327	bcl-6	100 (2.2)	56 (2.7)
21493970	hMSH6	137 (3.0)	18 (0.9)
21493971	PMS2	133 (2.9)	22 (1.1)
21492142	Cyclin D1	0 (0.0)	151 (7.2)
3002495	Desmin	132 (2.9)	18 (0.9)
3052827	CD8	140 (3.0)	8 (0.4)
3041284	CD10	86 (1.9)	60 (2.9)
3040360	Cytokeratin	79 (1.7)	67 (3.2)
3006921	Synaptophysin	110 (2.4)	32 (1.5)
3032734	MUM-1	48 (1.0)	55 (2.6)

### Database and Relationships Following the OMOP CDM Format

The CDM’s NOTE_NLP table is composed of three types of pathology reports; the major entities extracted from the immunohistochemical study reports and molecular study reports contain result summaries in the CDM’s MEASUREMENT table. Surgical site and cancer diagnosis information extracted from the surgical specimen pathology reports was constructed by populating the cancer diagnosis information in the patient’s CONDITION_OCCURRENCE table.

**Figure 5:**
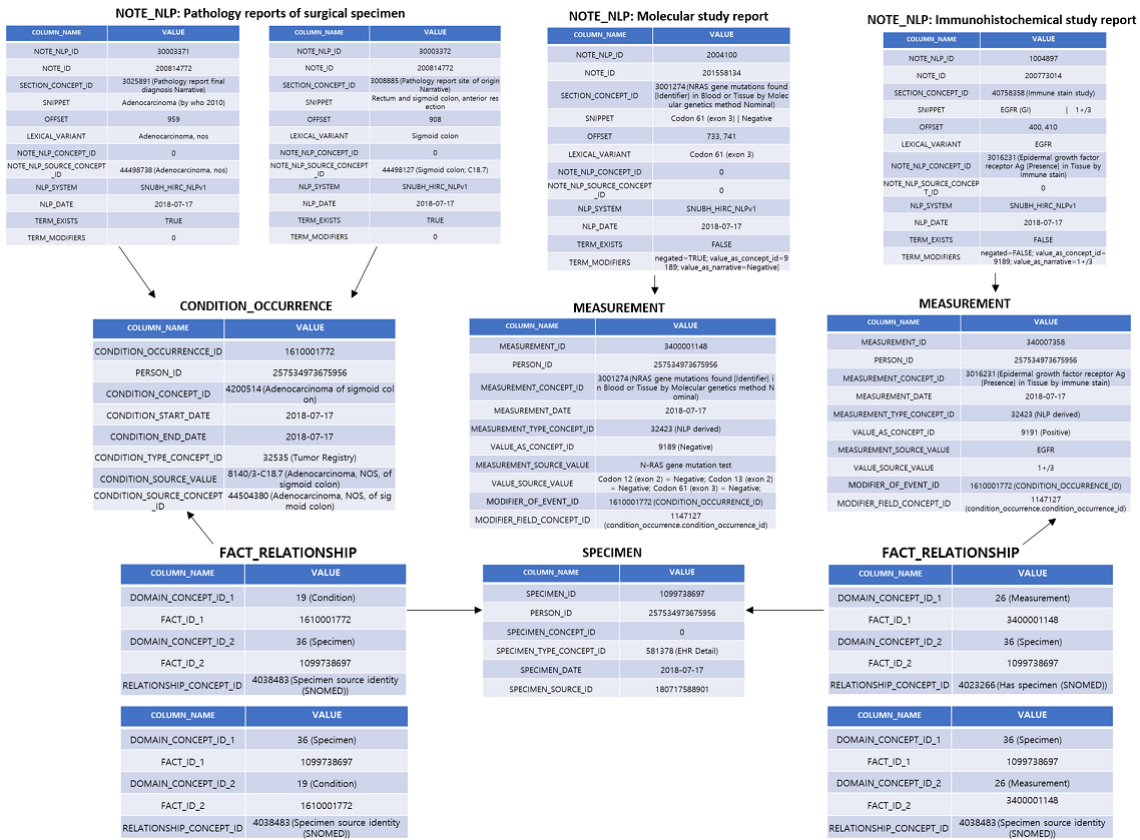
Overall data table relationships developed by the study.

The FACT_RELATIONSHIP table in the OMOP CDM contains records about the relationships between facts stored as records in any table of the CDM. Relationships can be defined between facts from the same domain or different domains. Examples of fact relationships include personal relationships (parent-child), care site relationships (hierarchical organizational structure of facilities within a health system), indication relationships (between drug exposures and associated conditions), usage relationships (of devices during the course of an associated procedure), or facts derived from one another (measurements derived from an associated specimen). To define the relationship between the MEASUREMENT, CONDITION_OCCURRENCE, and SPECIMEN tables, the FACT_RELATIONSHIP table of the CDM was populated (Figure 5).

In Figure 5, in the patient's surgical specimen pathology reports, records regarding the surgical site (the sigmoid colon) and diagnosis (“adenocarcinoma, nos”) are stored. These entities are delivered into the CONDITION_OCCURRENCE table, with cancer diagnosis information (adenocarcinoma of the sigmoid colon). Specimen information, including person_id and specimen_id during surgery, is stored in the SPECIMEN table. Here, the relationship definition is made using a FACT_RELATIONSHIP for the specimen with a diagnosis of CONDITION_OCCURRENCE. We used the “specimen source identity” concept to define and link the relationship between the two tables. The results are also stored in NOTE_NLP while performing the molecular study and immunohistochemical study tests with the specimen obtained from the operation. In our example, *NRAS* mutation test results in the molecular study report were negative; its details are “Codon 61 (exon 3)”. In addition, the information “NLP_derived N-RAS gene mutations found [identifier] in blood or tissue by molecular genetics method nominal” was updated in the MEASUREMENT table. In the same way, the immunohistochemical study test result for the EGFR test (“Epidermal growth factor receptor Ag [Presence] in Tissue by immune stain”), including the contents of the result “1+/3”, was saved in the NOTE_NLP table first; then, the NLP_derived EGFR positive summary is delivered to the MEASUREMENT table.

## Discussion

### Principal Findings

We demonstrate that we can store colon cancer–related textual entities processed through NLP, including those from concepts of standard vocabularies (eg, SNOMED CT, LOINC, and ICD-O), in CDM tables that can later be used for computational phenotyping. Our system can thus enable the development of new standard feature-based NLP systems and the reuse, portability, adaptation, and extension of other cancer-based reports. This is the first study to provide details on how to extract major entities and standardize each item in pathology examination reports. However, variations in text data used at different institutions complicate the application of our pattern-matching rule to other observational health care databases.

Concept recognition in biomedical text has been addressed by multiple systems such as MetaMap [17], the ConceptMapper Annotator of the Unstructured Information Management Architecture (UIMA) [18], and the Clinical Text Analysis and Knowledge Extraction System (cTAKES) [19]. MetaMap is a widely available program that provides access to concepts in the UMLS metathesaurus from biomedical text; it was introduced in an effort to improve biomedical text retrieval, specifically the retrieval of MEDLINE/PubMed citations. It also links the text of biomedical literature to knowledge, including synonymy relationships, embedded in the metathesaurus [17]. ConceptMapper Annotator was implemented as a UIMA component. It was designed to accurately map text onto controlled vocabularies, specified as dictionaries, including the association of any necessary properties from the controlled vocabulary as part of that mapping [18]. Although these tools allow the use of a custom dictionary, their approach to efficient task performance relies on the target text being grammatically well formed. Thus, these mappers rely on token-based matching, which is processed by sentence and phrase segmentation. Many of the abbreviations, typing errors, and implicit tables in clinical text blur sentence boundaries and make phrase segmentation challenging. Our proposed approach utilizes rule-based pattern matching and a set of terminology mappings among international standard vocabularies such as LOINC, SNOMED CT, and ICD-O that link both concepts and values.

Many previous studies have focused on developing new text processing technologies. For instance, Baghari et al [20] developed a UMLS-based biomedical semantic operator. Solt [21] developed a medication extraction system using combined conditional random fields and rule-based systems. NOBLE Coder [22] implements a general algorithm for matching terms to concepts from an arbitrary vocabulary set. Its developers benchmarked the system’s speed and accuracy against the Colorado Richly Annotated Full Text (CRAFT) [23,24] and Shared Annotated Resources (ShARe) [25] corpora as reference standards and compared it to other concept recognition systems for biomedical tasks. Moreover, the BioCreAtIvE (Critical Assessment of Information Extraction in Biology) competition for automated gene and protein name recognition consists of a community-wide effort to evaluate information extraction and text mining developments in the biological domain [26]. Mitsumori et al used the support vector machine algorithm as a learning method for gene and protein name recognition [27], investigating and evaluating the system’s performance when making partial dictionary pattern matches.

Regarding research on text engineering and CDMs, there has been a recent report of a cohort retrieval system that can execute textual cohort selection queries on both structured and unstructured EHR data using CDM data [28]. The system leveraged a combination of structured queries and information retrieval techniques in NLP results to improve cohort retrieval performance while adopting OMOP CDM to enhance model portability. The NLP component empowered by cTAKES was used to extract CDM concepts from textual queries, and a hierarchical index in Elasticsearch was generated to support CDM concept search using information retrieval techniques and frameworks [28].

Our study approach differs from those in previous studies based on the following characteristics. First, we analyzed three kinds of pathology reports and created pattern-matching rules for each. Second, we converted the colorectal cancer entities of colon cancer patients in our hospital into international standards to establish them in the OMOP CDM database. Third, a mapping dictionary was created to standardize terms for pathology reports; this is a reusable legacy asset and can be used in future studies. To our knowledge, this is the first time that relationships have been defined using FACT_RELATIONSHIP to link OMOP CDM to other tables; these are also recyclable assets that can be implemented in other studies.

In our study, text data extraction errors can occur during CDM conversion from the pathology reports, and errors may also be introduced in standard term mapping. To prevent pathology text data extraction errors, we had to check the correctness of all items to be extracted. Text extraction results of the NOTE_NLP table and the number of data stored in the CDM database table were compared to confirm errors in the CDM table. From randomly sampled documents, we measured the accuracy of each item extracted by comparing the original document with the NLP extraction result and confirmed that there were no errors. Standard term mapping errors were checked for by domain experts. Mapping errors were identified through expert reviews and double-checking by CDM researchers.

From this study, we derived the insight that domain experts such as pathologists and bioinformatics specialists are essential for accurate term mapping, as understanding genetic testing methods is required to examine pathology reports. Furthermore, it was difficult to automate mapping tasks because some terms could be mapped to many terms (1:N); another difficulty was confusion from incorrect standard term search results. Another important insight of this study is that the NOTE_NLP table does not have clear standard guidelines for modifiers, so further standardization studies on term modifiers are needed to express various kinds of information in the future.

There were several challenges faced in this study. More general application of the pattern-matching rules created in this study is difficult because report formats vary for every institution. In our hospital's surgical pathology report, the results of examination of the pathological tissues of various organs removed during surgery are written in one report. In this study, one surgical site and diagnostic information were extracted from one surgical pathology report, except for multi-organ results. This will have to be supplemented in further research. The text extraction process deals with the contents of one hospital's examination report and corresponding rules, which can make this system difficult to use in other studies; however, it is expected that the definition of the extracted entities and the standard term mapping data table will be available for other studies as well. Although text extraction is not universal, the mapping guidelines can be adjusted for reusability because it is possible to modify them to apply to other organizations. Since it is important to convert the data in the same standard concept_id in order to conduct multi-institution studies using CDMs, we expect that even small mapping sets that include the histologic type of invasive carcinoma, location information, and biomarker term information will be easier to map into standard terminology. In addition, this will be more useful because the data are from reports generated at a tertiary general hospital. It will be fully available as a reference for other institutions. Additionally, the textual components of cancer pathology in clinical documents are as diverse as the various noncolon cancer types, and they vary by location and over time with new forms and templates; therefore, further research is necessary for the normalization of different types of cancer records and for the establishment of an extended database. In addition, there are several ways to interpret the results of the immunohistochemical study report for colorectal cancer. In our study, biomarker results such as EGFR were mostly found in the results of the immunohistochemical study report of colorectal cancer. The EGFR expression and interpretation could be diverse based on pathologists' visual scoring or the fraction of carcinoma staining. In particular, the pathology department of our hospital chose the scoring method that maps EGFR results of 1+, 2+, and 3+ as “positive,” so we converted accordingly. If the scoring method should be subdivided into different biomarker results such as C-erbB2, this is a limitation of our research and something we should do in a future study.

In this study, we extracted fundamental text entities from pathological examination reports of patients with colon cancer and built a CDM database through terminology standardization and database definition. The preparation of pathology data for cancer and genome research, as well as various textual data that are currently recorded and managed, could lead to various elemental problems; further research on the CDM is expected to utilize much data. Our system can extract and store key features from unstructured text as NLP annotations by using the format defined by the OMOP CDM. The essential text entities from pathology reports were extracted, standardized, and deployed. Furthermore, more sophisticated preparation of the pathology data is needed for further research on cancer genomics, and various types of text narratives are areas for additional research on the use of data in the CDM.

### Limitations

This study could not be validated for an external organization for CDM. However, we attempted to build a consolidated CDM for dealing with narrative text, which enables researchers to more easily derive important information from unstructured data. Furthermore, with our concept-relation definition approach, other organizations can construct database structures. We hope to derive and apply more features of cancer-related text entities to utilize both clinical and omics data in further clinical studies based on this study.

## Appendix

Multimedia Appendix 1 Pseudo code for processing colorectal cancer pathology report.

Multimedia Appendix 2 Supplementary material for Immunochemistry concept mapping table.

## Abbreviations

CDM: common data model
cTAKES: Clinical Text Analysis and Knowledge Extraction System
EGFR: epidermal growth factor receptor
EHR: electronic health record
ICD-O: International Classification of Diseases for Oncology
LOINC: Logical Observation Identifiers Names and Codes
NLP: natural language processing
NOS: not otherwise specified
OHDSI: Observational Health Data Sciences and Informatics
OMOP: Observational Medical Outcomes Partnership
SNOMED: Systematized Nomenclature of Medicine
SNOMED CT: SNOMED Clinical Terms
SNUBH: Seoul National University Bundang Hospital
UIMA: Unstructured Information Management Architecture
UMLS: Unified Medical Language System
