# Supplementary material for: Transformation of Pathology Reports Into the Common Data Model With Oncology Module: Use Case for Colon Cancer
Source: J Med Internet Res. 2020 Dec 9;22(12):e18526. doi: 10.2196/18526 (PMC7758167; doi:10.2196/18526)
Supplement: Multimedia Appendix 1 [file jmir_v22i12e18526_app1.docx]

| **Report type** | **Target entity** | **Source text** | **Text processing rule** | **Results derived** |
| --- | --- | --- | --- | --- |
| Pathology reports of surgical specimen | Morphology | DIAGNOSIS : ADENOCARCINOMA | Extract diagnostic names following keywords ‘DIAGNOSIS:’  (E.g. DIAGNOSIS\S*[:]*(.*)) | ADENOCARCINOMA |
|  | Topology | SAMPLE NAME: Colon | Extract diagnostic names following keywords ‘SAMPLE NAME:’  (E.g. SAMPLE NAME\s*[:]*(.*)) | Colon |
| Pathology reports of immunohistochemical study and molecular study | Summary text | Summary: No altered expression of p53 | Extract items that exist behind ‘SUMMARY:’ and prior to ‘EXAMINER:’  (E.g. summary\s*[:]*(.*)[ EXAMINER:\|Note:]) | No altered expression of p53 |
|  | Biomarker names and results | p53 \| Negative | Extract left entity as biomarker name and right entity as its result value based on Vertical Bar (\|)  (E.g. ‘^(.*)\|’ , ‘.*\|(.*)$’) | p53,  Negative |

Table S1. Pathology report processing rules

*
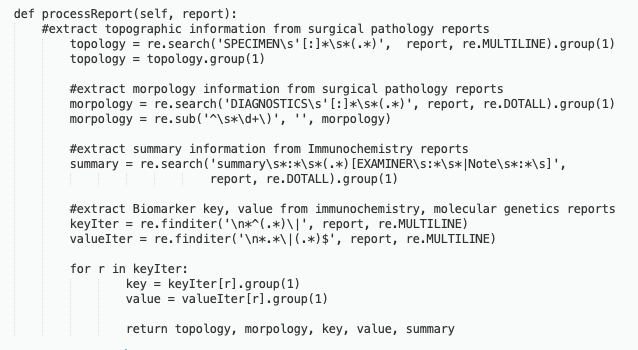
*Figure S1. Pseudo code for processing colorectal cancer pathology report
