# Supplementary material for: Transformation of Pathology Reports Into the Common Data Model With Oncology Module: Use Case for Colon Cancer
Source: J Med Internet Res. 2020 Dec 9;22(12):e18526. doi: 10.2196/18526 (PMC7758167; doi:10.2196/18526)
Supplement: Multimedia Appendix 2 [file jmir_v22i12e18526_app2.docx]

Table S2. Immunochemistry concept mapping table

| CONCEPT_ID | CONCEPT_NAME | DOMAIN_ID |
| --- | --- | --- |
| 3040758 | Actin smooth muscle Ag [Presence] in Tissue by Immune stain | Measurement |
| 3020498 | Alpha-1-Fetoprotein Ag [Presence] in Tissue by Immune stain | Measurement |
| 3032783 | Androgen receptor Ag [Presence] in Tissue by Immune stain | Measurement |
| 3051327 | BCL6 Ag [Presence] in Tissue by Immune stain | Measurement |
| 21493982 | BRAF protein [Presence] in Cancer specimen by Immune stain | Measurement |
| 3028984 | Beta catenin Ag [Presence] in Tissue by Immune stain | Measurement |
| 21492142 | CCND1 gene duplication [Presence] in Blood or Tissue by FISH | Measurement |
| 3041284 | CD10 Ag [Presence] in Tissue by Immune stain | Measurement |
| 3038382 | CD117 Ag [Presence] in Tissue by Immune stain | Measurement |
| 3031941 | CD138 Ag [Presence] in Tissue by Immune stain | Measurement |
| 3051613 | CD1a Ag [Presence] in Tissue by Immune stain | Measurement |
| 3026213 | CD20 Ag [Presence] in Tissue by Immune stain | Measurement |
| 3049424 | CD21 Ag [Presence] in Tissue by Immune stain | Measurement |
| 3035107 | CD227 Ag [Presence] in Tissue by Immune stain | Measurement |
| 3011037 | CD23 Ag [Presence] in Tissue by Immune stain | Measurement |
| 3027870 | CD3 Ag [Presence] in Tissue by Immune stain | Measurement |
| 3015513 | CD30 Ag [Presence] in Tissue by Immune stain | Measurement |
| 3051332 | CD31 Ag [Presence] in Tissue by Immune stain | Measurement |
| 3004224 | CD34 Ag [Presence] in Tissue by Immune stain | Measurement |
| 3053144 | CD4 Ag [Presence] in Tissue by Immune stain | Measurement |
| 3013639 | CD44 cells/100 cells in Blood | Measurement |
| 3041472 | CD45 Ag [Presence] in Tissue by Immune stain | Measurement |
| 3006739 | CD5 Ag [Presence] in Tissue by Immune stain | Measurement |
| 3006856 | CD56 Ag [Presence] in Tissue by Immune stain | Measurement |
| 3040355 | CD66e Ag [Presence] in Tissue by Immune stain | Measurement |
| 3048438 | CD68 Ag [Presence] in Tissue by Immune stain | Measurement |
| 3052827 | CD8 Ag [Presence] in Tissue by Immune stain | Measurement |
| 3052304 | CDX2 Ag [Presence] in Tissue by Immune stain | Measurement |
| 3049084 | Calretinin Ag [Presence] in Tissue by Immune stain | Measurement |
| 3022273 | Chromogranin Ag [Presence] in Tissue by Immune stain | Measurement |
| 21494129 | Chromosome region 6q22 rearrangements in Tissue by FISH | Measurement |
| 3040070 | Cytokeratin 20 Ag [Presence] in Tissue by Immune stain | Measurement |
| 3050376 | Cytokeratin 5/6 Ag [Presence] in Tissue by Immune stain | Measurement |
| 3040479 | Cytokeratin 7 Ag [Presence] in Tissue by Immune stain | Measurement |
| 3040360 | Cytokeratin AE1/AE3 Ag [Presence] in Tissue by Immune stain | Measurement |
| 3008089 | Cytomegalovirus Ag [Presence] in Tissue by Immune stain | Measurement |
| 43533931 | D2-40 Ag [Presence] in Tissue by Immune stain | Measurement |
| 21493968 | DNA mismatch repair protein Mlh1 [Presence] in Cancer specimen by Immune stain | Measurement |
| 21493969 | DNA mismatch repair protein Msh2 [Presence] in Cancer specimen by Immune stain | Measurement |
| 21493970 | DNA mismatch repair protein Msh6 [Presence] in Cancer specimen by Immune stain | Measurement |
| 43533934 | DOG1 Ag [Presence] in Tissue by Immune stain | Measurement |
| 3002495 | Desmin Ag [Presence] in Tissue by Immune stain | Measurement |
| 3016231 | Epidermal growth factor receptor Ag [Presence] in Tissue by Immune stain | Measurement |
| 4252545* | Epidermal growth factor receptor antagonist-containing product | Drug |
| 3041343 | Estrogen receptor Ag [Presence] in Tissue by Immune stain | Measurement |
| 3020364 | Glial fibrillary acidic protein Ag [Presence] in Tissue by Immune stain | Measurement |
| 3035754 | Glucagon Ag [Presence] in Tissue by Immune stain | Measurement |
| 3032704 | Granzyme B Ag [Presence] in Tissue by Immune stain | Measurement |
| 3019066 | HER2 Ag [Presence] in Tissue by Immune stain | Measurement |
| 3033296 | Herpes virus 8 latent nuclear Ag [Presence] in Tissue by Immune stain | Measurement |
| 3033051 | IgG subclass 4 Ag [Presence] in Tissue by Immune stain | Measurement |
| 2000000027** | Inhibin-alpha | Measurement |
| 4107216 | Interleukin-9 | Observation |
| 3015092 | Kappa light chains Ag [Presence] in Tissue by Immune stain | Measurement |
| 3046605 | Ki-67 nuclear Ag [Presence] in Tissue by Immune stain | Measurement |
| 3017020 | Lambda light chains Ag [Presence] in Tissue by Immune stain | Measurement |
| 4121191 | Langerhans cell histiocytosis stage | Measurement |
| 3032734 | MUM-1 Ag [Presence] in Tissue by Immune stain | Measurement |
| 3022366 | Microscopic observation [Identifier] in Blood or Marrow by Terminal deoxynucleotidyl transferase stain | Measurement |
| 21493971 | Mismatch repair endonuclease PMS2 [Presence] in Cancer specimen by Immune stain | Measurement |
| 3017031 | P53 protein Ag [Presence] in Tissue by Immune stain | Measurement |
| 43533932 | PAX8 Ag [Presence] in Tissue by Immune stain | Measurement |
| 42529558 | PD-L1 by clone 22C3 [Presence] in Tissue by Immune stain | Measurement |
| 21493983 | Phosphatase and tensin homolog (PTEN) protein [Presence] in Cancer specimen by Immune stain | Measurement |
| 3041608 | Progesterone receptor Ag [Presence] in Tissue by Immune stain | Measurement |
| 3007273 | Prostate specific Ag [Presence] in Tissue by Immune stain | Measurement |
| 4254723* | Proto-Oncogene Proteins c-mdm2 | Drug |
| 3016465 | S-100 Ag Ag [Presence] in Tissue by Immune stain | Measurement |
| 4255964* | Smad4 Protein | Drug |
| 3006921 | Synaptophysin Ag [Presence] in Tissue by Immune stain | Measurement |
| 3036403 | TP73L gene mutations found [Identifier] in Blood or Tissue by Molecular genetics method Nominal | Measurement |
| 4351425* | Thymidylate Synthase | Drug |
| 3038674 | Thyroid transcription factor 1 Ag [Presence] in Tissue by Immune stain | Measurement |
| 2000000020** | Transcription factor SOX-11 | Measurement |
| 718425* | Transcriptional Regulator ERG | Drug |
| 3025309 | Vimentin Ag [Presence] in Tissue by Immune stain | Measurement |
| 2000000026 | mucin 2 | Measurement |
| 3030110 | p16INK4a Ag [Presence] in Tissue by Immune stain | Measurement |

* Since it is a non-standard concept, mapping to the standard concept should be considered again in further.

** The concept is a custom concept newly defined through this study.
